# Case of Pneumothorax, Caused by a Fracture of the Clavicle

**Published:** 1838-08-15

**Authors:** 


*Case of Pneumothorax, caused by a fracture of the clavicle.*

Henry R——, forty-six years of age, born in Wales, a slater by trade, while engaged at his business, on the morning of the 7th of October, 1836, fell from the roof of a three story house, head foremost; his right arm struck the ground first, and he then fell on his back. He was brought to the hospital, immediately after the accident, with the following symptoms: extremities cold, pulse small and fluttering, 95 in the minute, anxious expression of countenance, great dyspnœa, and he complained of a feeling as "if he would suffocate." There was an oblique fracture of the right clavicle, the outer extremity of which was very much depressed, and driven backward, with ecchymosis over the lumbar vertebræ. The whole right side of the chest is very resonant on percussion, and the intercostal spaces very prominent; his sufferings were so great, that he could not permit any examination of the chest by auscultation. He could not bear the restraint of the dressings for the fractured clavicle; his arm was placed in a sling, sinapisms and warm tins applied to his extremities. In the evening he had re-acted slightly; complained of great pain in the right side; no respiration to be heard, but owing to his incessant groans, it was uncertain whether there might not be some action of the lung on that side. Tympanitic resonance of the whole of the right side anteriorly, below the third rib, and posteriorly below the fourth.

The action of the heart was very irregular and laboured; he expectorated a small quantity of bloody mucus, and passed some urine tinged with blood; pulse extremely feeble, 100; constant thirst; a short, dry, suppressed cough; ordered wine f℥ij. every hour, till relieved, and continue the warm applications. Cold water, which he craved very much, for drink.

*October 8th.* Dyspnœa diminished this morning; tongue red and dry; strong amphoric respiration heard over the right side of the chest, particularly distinct two inches below the clavicle; a flat sound on percussion, from the clavicle downwards, for two inches; he had an attack of pleurisy on this side two years ago, and the lung was confined by old adhesions. Pulse still very small, 96. No bloody expectoration, and the urine was perfectly limpid. Asked constantly for cold water, and refused all nourishment. Tr. opii gtt. xx. twice.

*October 9th.* Respiration much easier, lay on the back; right side more distended than yesterday, and as resonant on percussion as before; puerile respiration over whole of the left side. Complained of pain about the seat of fracture, and no where else; upon a minute examination, no other fracture can be detected than that of the clavicle, the outer extremity of which must have been driven backward and penetrated the lung. Amphoric respiration much more distinct under the second rib, than at any other part of the side. Tongue dry; pulse 96; no pain in urinating. In the evening, his respiration was more laboured, and he complained of severe pain in the right side, just under the nipple. He appeared to be slightly delirious; pulse 100, and very feeble. Ordered calomel gr. ¼, Dover's powder gr. iv., every three hours; dry cups to the side, followed by a warm poultice.

*October 10th.* Respiration more easy; no delirium; dull sound on percussion, at the lower part of the right side posteriorly, for two inches, showing the presence of a liquid; continued calomel and Dover's powder, and poultice.

*October 11th.* Symptoms much the same; great ecchymosis around the clavicle; he could bear no other application than a sling; he could not be made to keep the arm quiet; slight metallic tinkling to be heard after coughing.

*October 12th.* Improved; tongue moist and clean; pulse 96, and more volume; amphoric respiration still distinctly heard, but not so clearly as yesterday; the metallic tinkling continued; sound on percussion not so resonant as before, and no expectoration, and but slight cough.

*October 21st.* He has continued gradually improving since the last report; the metallic tinkling has disappeared; the flatness on percussion has gradually increased; strong bronchial respiration to be heard under the clavicle; from his obstinacy in constantly moving his arm, it has been confined with a glove and padlock, as he said that he was quite well, and would allow nothing to be kept on that he could remove.

*October 26th.* Has had erysipelas over the seat of fracture, which terminated in the formation of an abscess; upon opening it, a large quantity of healthy pus was discharged, and the bone could be felt covered with granulations.

*November 28th.* A silk ligature was passed between the ends of the bone, to cause long union; respiration was now puerile on the left side, and very feeble on the right, except under the clavicle, where slight bronchial respiration could still be heard. There was also flatness on percussion over the whole right side of the chest.

*December 21st.* He has improved gradually since the last report; the union of the fracture perfectly firm; but in consequence of his great restlessness, he has considerable deformity. The right side of the chest has contracted so as to be less by two inches in circumference than the opposite side; still a dull sound on percussion over it, and the respiration slightly bronchial, under the clavicle and posteriorly. Being anxious to return home, he was discharged this morning.

The pointed extremity of the fracture, in consequence of the direction in which he fell, must have been driven downwards and backwards, and penetrated the apex of the lung. There was a case of fractured clavicle admitted two years ago, produced by a fall upon the hand, which was followed by emphysema, showing the existence of a wound of the lung; it was not followed by any bad symptoms, and a cure took place under the ordinary treatment.

